# Overbaked: assessing and predicting acute adverse reactions to Cannabis

**DOI:** 10.1186/s42238-019-0013-x

**Published:** 2020-01-02

**Authors:** Emily M. LaFrance, Amanda Stueber, Nicholas C. Glodosky, Dakota Mauzay, Carrie Cuttler

**Affiliations:** 1 Department of Psychology, Washington State University, P.O. Box 644820, Pullman, WA 99164-4820 USA; 2 Translational Addiction Research Center, Washington State University, Pullman, WA USA

**Keywords:** Cannabis use, Adverse reactions, Side effects, Anxiety, Paranoia

## Abstract

**Background:**

Trends toward legalizing cannabis may increase experimentation with the drug among less experienced users with limited knowledge of possible adverse reactions. This study explores the prevalence, frequency, and levels of distress produced by various acute adverse reactions to cannabis, as well as predictors of these reactions.

**Methods:**

The Adverse Reactions Scale (ARS) was created and administered to a large sample of undergraduate college students (*n* = 999) who were predominantly white (> 70%), female (> 70%), recreational (> 90%) cannabis users. The ARS was administered in an anonymous online survey measuring demographics, cannabis use patterns, cannabis use motives, personality, and negative affect.

**Results:**

The most prevalent adverse reactions to cannabis were coughing fits, anxiety, and paranoia, which > 50% of the sample reported experiencing. The most frequently occurring reactions were coughing fits, chest/lung discomfort, and body humming, which occurred on approximately 30–40% of cannabis use sessions. Panic attacks, fainting, and vomiting were rated as the most distressing, with mean ratings falling between “moderately” and “quite” distressing. Multiple regression analyses revealed that lower frequency of cannabis use predicted increased frequency of adverse reactions. Symptoms of cannabis use disorder, conformity motives, and anxiety sensitivity were significant predictors of both the prevalence of, and distress caused by, adverse reactions.

**Conclusions:**

Relative to past research, this study provides a more comprehensive account of possible adverse reactions to cannabis, and individual difference variables that predict these reactions. This study has implications for inexperienced cannabis users, as well as medical professionals and budtenders who provide information about cannabis use.

## Background

Currently, 33 states and the District of Columbia (D.C.) have legalized medical cannabis, while 11 states, D.C., and Canada have legalized recreational cannabis. Increased access to legal cannabis in North America may increase experimentation with the drug among less experienced/naïve users who may have limited knowledge of the possible adverse effects of cannabis. This problem is further compounded by limited research on individual difference variables which may increase one’s propensity to experience such adverse effects. As such, individuals with limited cannabis use experience may be unaware that they could be at increased risk of experiencing negative acute effects of cannabis. Similarly, health care professionals making recommendations for medical cannabis use could be unaware that their patients may be at elevated risk of adverse reactions to cannabis.

Some of the most common adverse reactions to cannabis intoxication include paranoia (Arendt et al. 2007), dry mouth (Sexton et al. 2019), memory problems (Sexton et al. 2019), and an altered sense of perception/time (Arendt et al. 2007; Sexton et al. 2019). Less commonly documented acute adverse reactions include hallucinations (Arendt et al. 2007; Sexton et al. 2019), sadness/depression (Arendt et al. 2007), dizziness (Sexton et al. 2019), confusion (Sexton et al. 2019), and lack of coordination (Sexton et al. 2019). However, anecdotal evidence suggests that there may be a broader range of acute adverse reactions to cannabis, including dissociation, coughing fits, vomiting, and other symptoms (Mullaney 2018; Rahn 2016; WebMD 2018) which have not received sufficient attention in past research. A more comprehensive understanding of the different types of negative acute reactions to cannabis would benefit both the scientific and medical communities, as well medical and recreational cannabis users, who may be at increased risk of experiencing some of these under-investigated adverse reactions to cannabis.

There has been some indication that there are individual differences in the chronic psychophysiological effects of cannabis (Atakan et al. 2013; Volkow et al. 2014). For instance, past research has established that psychotic symptoms (Atakan et al. 2013), coping-related motives for cannabis use (e.g., Moitra et al. 2015; Spradlin and Cuttler 2019), and adolescent-onset cannabis use (Volkow et al. 2014) predict negative consequences associated with chronic cannabis use. This suggests that some cannabis users may be more prone to experience negative side effects of chronic cannabis use (e.g., impaired cognitive functioning, cannabis abuse/addiction, and increased risk of mental illness).

In contrast, fewer studies have focused on negative *acute* reactions to cannabis (Arendt et al. 2007; Sexton et al. 2019; Vadhan et al. 2017). However, a small body of research indicates that higher levels of depression and anxiety (Arendt et al. 2007) predict negative mood states and paranoia during periods of acute intoxication, while a genetic predisposition toward psychotic symptoms predicts transient psychotic-like states during intoxication (Vadhan et al. 2017). Additionally, one recent study found that younger cannabis users (regardless of medical/recreational user status) were more likely to report various undesirable acute effects of cannabis intoxication than older individuals (50+). Further, recreational users (regardless of age) were also more likely to report undesirable effects than medical cannabis users (Sexton et al. 2019). However, to our knowledge, no previous studies have examined personality, cannabis use patterns (e.g., frequency, quantity, age of onset), or motives for cannabis use as potential predictors of acute adverse reactions to cannabis.

### Purpose and hypotheses

The present study was designed to assess the prevalence and frequency of a wide range of acute adverse reactions to cannabis as well as the level of distress associated with each of these reactions. We further sought to explore individual difference variables that might predict these adverse reactions. We expected that anxiety, coughing fits, and paranoia would be among the most common acute adverse reactions to cannabis and that fainting, hallucinations, paranoia, and panic attacks would be some of the most distressing reactions. Based on previous research (Arendt et al. 2007), we also hypothesized that less frequent cannabis use and higher levels of negative affect (e.g. anxiety, anxiety sensitivity, depression, and neuroticism) would predict higher frequency of adverse reactions to cannabis, as well as higher levels of distress produced by these reactions.

## Methods

### Procedures

A large sample of undergraduate students was recruited from the Washington State University Department of Psychology’s subject pool between August of 2018 and May of 2019, using the university SONA system, which is an online system for advertising and assigning credit to participants. This system further ensures that participants only complete the survey once. Participants elected to complete this study by selecting it from a list of ongoing studies. After providing informed consent, participants completed an anonymous online survey that took approximately 30 min to complete (see Measures below). No responses on the survey were forced choice. For their time, participants were compensated with course credit, which could be used towards eligible Psychology courses. The Office of Research Assurances deemed this project minimal-risk research, and therefore exempt from the need for review by the Institutional Review Board.

### Participants

The total sample comprised 1588 undergraduate students. A student sample was selected because cannabis use is most frequent among young adults (Substance Abuse and Mental Health Services Administration [SAMHSA] 2016). In order to be included, participants had to be at least 18 years of age and fluent in English. All participants met these criteria. The only exclusion criteria were reporting never having used cannabis and evidence of random responding, which was measured by interspersing the 10 items from the deviant responding validity subscale of the Psychopathic Personality Inventory (PPI; Lilienfeld and Andrews 1996) throughout the survey. A total of 230 participants (14.5% of the total sample) endorsed five or more of the PPI items in an aberrant manner and were excluded from all analyses.^1^ Additionally, 319 participants (23.5% of the remaining sample) reported never using cannabis and were excluded. Finally, 40 individuals (3.9% of the remaining sample) failed to indicate whether or not they had ever used cannabis and had missing data on a significant portion of the other measures and were also excluded from subsequent analyses. As such, the final sample size was 999. Demographic characteristics and cannabis use patterns for the final sample are provided in Table 1.

**Table 1:** Demographic and cannabis use characteristics of 999 college undergraduate students

	Mean (Standard Error [SE])	Range
Age (years)	20.94 (0.14)	18–61
Education (years of university)	2.16 (0.04)	1–6
Cannabis Use in Past Month (days)	8.42 (0.33)	0–31
Cannabis Use in Past Week (days)	2.02 (0.08)	0–7
Age of First Cannabis Use (years)	16.71 (0.07)	1–34
Duration of Cannabis Use (years)	3.27 (0.11)	0–35
Quantity of Cannabis Used per Week (grams)	2.17 (0.14)	0–28
Cannabis Potency (% THC)	4.63 (0.08)	1–7
% of Respondents		
White	72.7%	
Female	72.7%	
Medical Cannabis Use	5*.*2%	
Primary Method of Administration Inhalation	73.4%	
Primary Method of Administration Edibles	11.4%	

The scale used to measure cannabis potency/%THC was as follows 1 = 0–4%, 2 = 5–9%, 3 = 10–14%, 4 = 15–19%, 5 = 20–24%, 6 = 25–30%, 7 = > 30%

### Measures

*Demographics.* A short demographics questionnaire was used to assess age, gender identity, education, and ethnicity.

*Adverse Reactions to Cannabis.* In order to assess the frequency and prevalence of various adverse reactions to cannabis, we created the Adverse Reactions Scale (ARS) by consulting existing empirical literature (Atakan et al. 2013; Chabrol et al. 2005; Moitra et al. 2015; Sexton et al. 2019; Vadhan et al. 2017), popular media (Mullaney 2018; Rahn 2016; WebMD 2018), and a small number of experienced recreational and medical cannabis users. The ARS contains a list of 26 different adverse reactions that are listed in Table 2. Participants were asked to indicate whether or not they had ever experienced each reaction when they were high on cannabis using a binary yes/no scale. For each endorsed reaction, they were further asked to rate how distressing they found it to be, using a 5-point Likert scale (0 = Not at all distressing, 1 = Slightly distressing, 2 = Moderately distressing, 3 = Quite distressing, 4 = Severely distressing). A subset of participants (*n* = 370)^2^ were further asked to indicate the relative frequency at which they experienced each reaction when using cannabis (i.e., the percent of cannabis use sessions during which they experienced each adverse reaction). Skip logic was used so that individuals who did not endorse experiencing a particular adverse reaction to cannabis were not prompted to answer questions about its frequency or associated levels of distress. The complete ARS is available in Additional file 3. A total of four scores were computed: 1) prevalence, which reflects the percentage of the sample who indicated they had experienced the reaction at least once, 2) frequency, which reflects the mean percent of cannabis use sessions during which they experienced each reaction, 3) distress, which represents average distress ratings, and 4) number of different symptoms experienced, which reflects the total number of endorsed symptoms. Cronbach’s alpha values were .90 for prevalence, .75 for frequency, and .99 for distress. Each of the three ARS outcome variables were normally distributed, with acceptable skew and kurtosis values (i.e. values smaller than +/− 2.0; George and Mallery 2010).

**Table 2:** Self-reported frequency and distress associated with adverse reactions to cannabis

ARS Symptom	Prevalence^1^	Frequency^2^ (SE)	Distress^3^ (SE)
Coughing Fit	62.23%	41.49 (2.28)	1.10 (0.04)
Anxiety	53.19%	24.79 (2.16)	2.10 (0.05)
Paranoia	50.30%	25.21 (2.38)	2.07 (0.05)
Off-balance/Unsteady	48.88%	22.14 (2.37)	0.96 (0.05)
Light-headed/Head Rush	47.22%	23.72 (2.47)	1.03 (0.05)
Dissociation	41.19%	26.32 (2.79)	1.31 (0.07)
Dizziness	39.31%	22.49 (2.85)	1.28 (0.06)
Body Humming	38.58%	29.98 (3.01)	0.73 (0.06)
Racing Heart	38.16%	28.17 (2.66)	1.73 (0.06)
Feeling Out of Control	37.01%	17.63 (2.46)	1.95 (0.07)
Numbness	33.37%	25.98 (2.71)	0.75 (0.06)
Chest/Lung Discomfort	30.66%	31.69 (3.01)	1.53 (0.07)
Nausea	28.06%	10.65 (2.01)	1.71 (0.07)
Tunnel Vision	24.92%	19.48 (3.33)	1.41 (0.09)
Panic Attack	23.12%	15.03 (2.88)	2.71 (0.09)
Trouble Breathing	23.06%	17.19 (2.61)	1.98 (0.08)
Migraine/Headache	21.12%	11.58 (2.18)	1.26 (0.07)
Vomiting	18.44%	6.11 (1.69)	2.28 (0.10)
Visual Hallucinations	17.21%	13.87 (3.76)	1.63 (0.11)
Auditory Hallucinations	17.07%	21.05 (3.96)	1.38 (0.10)
Seeing Black Spots	16.26%	14.30 (2.94)	1.72 (0.11)
Hot Flashes	15.89%	18.77 (4.14)	1.38 (0.10)
Heart Palpitations	12.72%	21.84 (4.60)	1.86 (0.12)
Cold Sweats	11.09%	8.84 (2.65)	1.43 (0.12)
Other Hallucinations	6.70%	21.67 (7.01)	1.47 (0.20)
Fainting / Passing Out	6.61%	15.70 (6.32)	2.39 (0.19)

^1^ Percentage of participants who endorsed each reaction (*N* = 999)

^2^ Mean percent of cannabis use sessions during which each reaction was experienced (with standard

errors in parentheses; *N* = 370)

^3^ Mean distress rating associated with each reaction (with standard errors in parentheses; *N* = 999)

#### Cannabis use patterns

The Daily Sessions, Frequency, Age of Onset, and Quantity of Cannabis Use Inventory (DFAQ-CU; Cuttler and Spradlin 2017) was used to assess cannabis use patterns and determine participants’ eligibility to participate in the study. The DFAQ-CU contains 33 items and six subscales (daily sessions, frequency, age of onset, quantity of cannabis, quantity of concentrates, and quantity of edibles used). For the present study, results from only the first four subscales are presented, because the majority of participants (73.4%) indicated that they primarily inhaled cannabis flower, which would reduce power to detect significant relationships with the concentrates and edibles quantity factors. Indeed, exploratory analyses failed to reveal any significant correlations between quantity of concentrates or edibles used, and any of the outcome variables on the ARS (see Additional file 2: Table S2). Scores were computed by averaging the standardized items within each subscale. The psychometric properties of this inventory have been previously established. More specifically, a factor analysis found that Cronbach’s alpha coefficients ranged from .69 to .95 for the six subscales (Cuttler and Spradlin 2017). For the current sample, Cronbach’s alpha was .74 for daily sessions, .70 for frequency, .72 for age of onset, and .54 for quantity of cannabis used. The lower Cronbach’s alpha value for the quantity subscale is likely because this factor score is comprised of only three raw scores.

#### Cannabis use disorder

The Cannabis Use Disorder Identification Test-Revised (CUDIT-R; Adamson et al. 2010) was administered to assess symptoms of cannabis use disorder. The CUDIT-R is an 8-item self-report scale containing various symptoms of cannabis use disorder. Each item is measured using a 5-point scale. These items were averaged and as such total scores for the CUDIT-R could range from 0 to 4. In past research, the CUDIT-R has demonstrated sound psychometric properties, with a Cronbach’s alpha value of .91, test retest reliability value of .85, and discriminant validity value of .93 (Adamson et al. 2010). In the present sample, Cronbach’s alpha was .77.

#### Cannabis use motives

The Marijuana Motives Measure (MMM; Benschop et al. 2015) was administered to assess common motives for cannabis use. The MMM contains 29 items which assess six different motives for using cannabis: coping (e.g., “to forget my worries”), enhancement (e.g., “because it gives me a pleasant feeling”), social (e.g., “it makes social gatherings more fun”), conformity (e.g., “to be liked”), expansion (e.g., “to understand things differently”), and routine (e.g., “out of habit”). Responses were recorded on a 5-point Likert scale (1 = Almost never/never, 2 = Some of the time, 3 = Half of the time, 4 = Most of the time, and 5 = Almost always/always). The average of each subscale was calculated, with higher scores representing stronger endorsement of the motive. The MMM has demonstrated good internal consistency with Cronbach’s alpha values ranging from .72 to .85 (Benschop et al. 2015). In the current sample, Cronbach’s alpha values for the six subscales ranged from .79 to .93.

#### Depression, anxiety, stress

The Depression Anxiety Stress Scale (DASS-21) consists of 21 items and has three subscales which measure symptoms of depression, anxiety, and stress. Participants were asked to rate the extent to which they have experienced various symptoms of depression, anxiety, and stress over the past week. The DASS-21 was scored by averaging the items on each subscale. Therefore, possible scores range from 0 to 3, with higher scores on each subscale representing greater symptom severity. The DASS-21 has demonstrated sound psychometric properties in previous research, with test-retest reliability values ranging from .82 to .97 and concurrent validity values between .40 to .65 (Osman et al. 2012). In the current sample, Cronbach’s alpha values were .90 for depression, .82 for anxiety, and .86 for stress.

#### Anxiety sensitivity

The Anxiety Sensitivity Index (ASI; Peterson and Heilbronner 1987) was administered to measure the degree to which participants believe that physical symptoms of anxiety are negative or catastrophic. Participants were asked to self-report the degree to which they agree with 16 statements such as, “It is important for me not to appear nervous” on a 5-point Likert scale (0 = Very little to 4 = Very much). The ASI was scored by averaging all items and as such possible scores range from 0 to 4, with higher scores representing higher anxiety sensitivity. The ASI has demonstrated sound psychometric properties in past research, with internal reliability values ranging from .85 to .88 (Peterson and Heilbronner 1987). For the present sample, Cronbach’s alpha was .89.

#### Personality

The Neuroticism, Extraversion, Openness to Experience Five Factor Inventory, (NEO-FFI; McCrae and Costa 2010) was used to measure the Big 5 personality traits (i.e., neuroticism, extraversion, openness to experience, agreeableness, conscientiousness). Participants rated the extent to which they endorsed various items which measured these five traits on a 5-point Likert scale (0 = Strongly disagree, 1 = Disagree, 2 = Neutral, 3 = Agree, 4 = Strongly agree). The NEO-FFI has demonstrated sound reliability and validity, with validity coefficients ranging from .86 to .92, and test-retest reliability coefficients ranging from .66 to .92 across the five subscales (McCrae and Costa 2010). For the present sample, Cronbach’s alpha was .84 for neuroticism, .80 for extraversion, .51 for openness to experience,^3^ .73 for agreeableness, and .82 for conscientiousness.

### Data analysis

All data were analyzed using SPSS version 25. The data were screened for outliers, and raw scores greater than 3.29 standard deviations away from the mean were trimmed to one raw score value above or below the nearest non-outlying score. A total of 84 outliers (0.001% of the total data analyzed) were trimmed in the dataset.

Prevalence of each reaction was assessed by computing the percentage of the sample who endorsed each reaction. Frequency was determined by computing the mean percentage of cannabis use occasions during which each reaction was experienced. Levels of distress associated with each reaction were assessed by computing the mean distress rating for each reaction. The prevalence, frequency, and mean levels of distress associated with each reaction are provided in Table 2. The total number of different adverse reactions experienced was also computed by tallying the number of different adverse reactions endorsed.

A series of standard multiple regression analyses were conducted to examine the relationships between each of the specific predictors and three ARS outcome variables (number of different reactions experienced, average frequency of reactions, and average distress produced by reactions), while controlling for all other predictors in the model. The predictor variables that were included, the standardized beta values, and the standard errors from these multiple regression analyses are reported in Table 3. A Bonferroni corrected alpha level of .017 (alpha = .05/3 = .017) was used to help control for inflation in familywise-Type I error for these three regression analyses.

**Table 3:** Multiple Regression Exploring Predictors of Self-Reported Adverse Reactions to Cannabis

Predictor	# Different Reactions (SE)	Overall Frequency (SE)	Overall Mean Distress (SE)
Daily Sessions of Cannabis Use	.03 (0.43)	.002 (2.44)	.003 (.06)
Frequency of Cannabis Use	−.15 (0.50)	−.43** (2.63)	−.10 (.07)
Age of Onset of Cannabis Use	−.05 (0.31)	−.09 (1.86)	−.11 (.05)
Quantity of Cannabis Use	−.02 (0.35)	.08 (1.81)	.05 (.05)
Cannabis Use Disorder	.18** (0.45)	.09 (2.54)	.17* (.07)
Coping Motives	−.08 (0.27)	−.10 (1.53)	−.14 (.04)
Enhancement Motives	.05 (0.28)	−.03 (1.46)	−.002 (.04)
Social Motives	−.08 (0.27)	.10 (1.46)	−.11 (.04)
Conformity Motives	.12* (0.38)	.02 (2.07)	.21** (.06)
Expansion Motives	.05 (0.24)	−.03 (1.28)	−.02 (.04)
Routine Motives	.05 (0.31)	−.06 (1.74)	−.04 (.05)
Openness to Experience	.08 (0.52)	−.08 (3.14)	−.04 (.07)
Conscientiousness	−.04 (0.44)	−.10 (2.27)	.11* (.06)
Extraversion	.04 (0.48)	.11 (2.61)	−.05 (.07)
Agreeableness	.11* (0.50)	.06 (2.80)	.08 (.07)
Neuroticism	.14 (0.51)	.19 (2.71)	.13 (.07)
Anxiety Sensitivity	.15* (0.39)	−.07 (2.18)	.22** (.06)
Depression	−.03 (0.53)	−.04 (3.10)	.03 (.08)
Anxiety	−.01 (0.61)	.01 (3.60)	−.07 (.09)
Stress	.05 (0.59)	.14 (3.15)	−.01 (.08)
Age	−.07 (0.08)	.12 (0.52)	.04 (.01)
Gender	.07 (0.53)	.09 (2.77)	.09 (.08)

*Note:* Standardized Beta values are presented with standard errors in parentheses

* denotes *p* < .017 (Alpha level calculated using a Bonferroni correction for the three multiple regression analyses computed (α/n = .05 / 3 = .017)).

** denotes *p* < .001

## Results

### Prevalence, frequency, & distress associated with specific adverse reactions to Cannabis

A complete breakdown of the prevalence, average frequency and severity of distress associated with each adverse reaction are presented in Table 2. Additional file 2: Table S2 displays the bivariate correlations between all of the predictor and outcome variables.

#### Prevalence of adverse reactions

The three most prevalent adverse reactions to cannabis intoxication were coughing fits, anxiety, and paranoia. The three least common reactions were fainting/passing out, other (non-auditory/visual) hallucinations, and cold sweats.

#### Frequency of adverse reactions

A subset of participants (*n* = 370) were asked to report the approximate frequency at which they experienced each individual adverse reaction. Coughing fits, chest/lung discomfort, and body humming were the three most frequently experienced reactions to cannabis.

#### Distress

The most distressing adverse reactions were panic attacks, fainting/passing out, and vomiting, while the least distressing reactions were body humming, numbness, and feeling off balance/unsteady.

### Predictors of adverse reactions

#### Predictors of the number of different adverse reactions

The results of a multiple regression analysis revealed that the set of 22 predictors accounted for 15.7% of the total variability in number of different adverse reactions experienced, which was statistically significant, *F* (24, 637) = 4.94, *p* < .001. More specifically, as shown in Table 3, symptoms of cannabis use disorder, conformity motives, agreeableness, and anxiety sensitivity each accounted for a significant portion of unique variance in the number of different adverse reactions to cannabis experienced.

#### Predictors of the frequency of adverse reactions

The results of a standard multiple regression analysis revealed that collectively the set of predictors accounted for 25.7% of the total variance in frequency of adverse reactions to cannabis, which was statistically significant, *F* (24, 208) = 3.00, *p* < .001. However, only frequency of cannabis use accounted for a significant portion of unique variance in the frequency of adverse reactions to cannabis (see Table 3).

#### Predictors of distress associated with adverse reactions

The final regression model revealed that the set of predictors accounted for 17.8% of the variance in distressed produced by adverse reactions to cannabis, *F* (24, 581) = 5.23, *p* < .001. Cannabis use disorder symptoms, conformity motives, conscientiousness, and anxiety sensitivity were all found to be significant predictors of levels of distress produced by adverse reactions to cannabis (see Table 3).

## Discussion

This study builds upon a small previous literature documenting the prevalence and frequency of various adverse reactions to cannabis. Specifically, Sexton et al. (2019) recently asked a large sample (> 2900) of cannabis users to indicate which of a list of acute effects (positive and adverse) they experience when they are intoxicated on cannabis. Their results indicated that dry mouth was the most prevalent adverse reaction to cannabis (63% of their sample endorsed experiencing this reaction), followed by memory problems (42.2%), tiredness (45.9%), and altered sense of time (37.6%). Further, they found that paranoia (14.5%), anxiety (8.6%), lung discomfort (7.6%), dizziness (5%) and hallucinations (3.8%), were less commonly endorsed adverse reactions. In contrast, findings from the present study indicate that paranoia (50.3%), anxiety (53.2%), coughing fits (62.2%), chest/lung discomfort (30.7%), dizziness (39.3%), and hallucinations (17%) are experienced by a larger percentage of cannabis users, with anxiety, paranoia, and dizziness reported on approximately 25% of cannabis use sessions, coughing fits reported on approximately 40% of all sessions, chest/lung discomfort reported on approximately 30% of cannabis use sessions, and auditory hallucinations experienced on approximately 20% of these sessions. The discrepancies in these findings may reflect differences in the methods of assessing these reactions and/or the samples surveyed. Specifically, Sexton et al. (2019) simply asked respondents to indicate which reactions they experience while intoxicated while we asked respondents to indicate which reactions they *had ever experienced* while intoxicated. As such, participants in Sexton’s (2019) study may have reported on which they more commonly experience rather than which they had ever experienced. Further, the participants in Sexton’s study were a broader community sample of recreational, medical, and mixed (recreational and medical) users with a wider range of ages than participants in the present study (which focused predominantly on young recreational users). It is possible that older, more experienced cannabis users experience fewer adverse reactions. Indeed, their findings did indicate that older users and medical users were less likely to report adverse reactions than younger recreational users (Sexton et al. 2019). Collectively these findings suggest that younger, less experienced, recreational cannabis users may be more prone to experiencing adverse reactions to cannabis.

Arendt et al. (2007) measured the frequency of various reactions (positive and negative) to acute intoxication in a relatively small sample of 119 cannabis-dependent individuals. Their results indicated that altered perceptions, delusions, slower movements, and confusion were the most frequent adverse reactions to cannabis intoxication (with mean ratings between sometimes and often). In contrast, hallucinations, self-reported feelings of anxiety, and sadness/depression during intoxication were infrequent in their sample (with mean ratings indicating these reactions are rarely experienced). The present study focused on largely different reactions than theirs and results from our study indicate that anxiety and hallucinations are experienced more frequently (on approximately 20–25% of all cannabis use sessions). Once again, it is likely that differences in the samples used in these studies accounts for differences in our findings. The cannabis-dependent individuals in Arendt et al.’ (2007) study likely have more experience with cannabis and as such may be more tolerant to its potentially adverse effects.

This study extends upon previous research that has focused exclusively on prevalence (Sexton et al. 2019) and frequency (Arendt et al. 2007) of adverse reactions by further indicating which reactions are perceived as the most and least distressing by cannabis users. It is worth noting that overall, even the most distressing reactions to cannabis were only rated between ‘moderately’ and ‘quite distressing,’ on average, suggesting that cannabis users do not, in general, find acute adverse reactions to cannabis to be severely distressing. Further, feelings of body humming, numbness, and unsteadiness were rated as the least distressing, with participants on average indicating that these specific reactions were between ‘not at all’ to ‘slightly’ distressing. As such, these reactions to cannabis intoxication may not actually be interpreted as adverse by cannabis users and could be removed from the ARS for future studies. In contrast, paranoia and anxiety were rated as ‘moderately distressing’ on average and were also highly prevalent and frequent. Consequently, these potential adverse reactions may be of more concern, and should be emphasized as the most common distressing reactions.

The results of the standard regression analysis using all 22 predictors to predict the frequency of adverse reactions to cannabis revealed that only frequency of cannabis use accounted for a significant portion of unique variance in this outcome. The regression coefficient was moderately sized and negative, indicating that more frequent cannabis use is associated with less frequent adverse reactions to cannabis. This indicates that regular cannabis users, who are more accustomed to the acute effects of cannabis, experience adverse reactions during a smaller percentage of their cannabis use sessions. This may be due in part to the development of tolerance to the intoxicating effects of cannabis, which occurs with regular cannabis use. Indeed, past research has also found that regular cannabis users can develop tolerance to the cognitively impairing effects of cannabis (Colizzi and Bhattacharyya 2018; D’Souza et al. 2008; Ramaekers et al. 2016), and partial tolerance to the anxiogenic, psychotomimetic, and cardiac effects of cannabis also occurs with regular exposure (Colizzi and Bhattacharyya 2018). Additionally, experienced cannabis users are likely better accustomed to self-titrating their cannabis ingestion when they have reached their desired level of intoxication, whereas less frequent users may not properly self-titrate. Alternatively, our results may indicate that individuals who frequently experience adverse reactions may find cannabis less desirable and choose to use the drug less often.

Symptoms of cannabis use disorder, conformity motives, and anxiety sensitivity, each accounted for significant portions of unique variance in the number of different adverse reactions to cannabis experienced, as well as in the levels of distress produced by these reactions. Each of these regression coefficients was small and positive, indicating that problematic cannabis use, cannabis use motivated by a desire to fit in with peers, and higher levels of anxiety sensitivity are associated with reporting a greater number of different adverse reactions, and with experiencing higher levels of distress during these reactions.

By definition, individuals with more severe symptoms of cannabis use disorder continue to use cannabis despite experiencing negative consequences associated with their use (e.g. problems with memory, impairment in functioning; Adamson et al. 2010). Our results appear to indicate that individuals with more severe symptoms of cannabis use disorder also might continue to use cannabis despite experiencing numerous distressing acute adverse reactions to cannabis, whereas individuals with lower levels of these symptoms may be more likely to cease cannabis use after experiencing a variety of distressing adverse reactions to cannabis.

Individuals using cannabis to conform to peer pressure may be less experienced users who are not accustomed to, nor enjoy, the acute effects of cannabis intoxication, but use cannabis to fit in with their peers. This seems to suggest that one’s expectations for the effects of cannabis and/or their mindset prior to cannabis use may shape the valence of their experience while intoxicated and that those using specifically for conformity motives are more likely to experience a variety of acute, distressing reactions to cannabis.

Individuals with higher levels of anxiety sensitivity also reported a greater variety of adverse reactions, and experienced higher levels of distress associated with these adverse reactions. Anxiety sensitivity refers to the tendency to fear anxiety-related sensations and to catastrophize about the meaning of these sensations. Acute cannabis intoxication can cause elevated heart rate, anxiety, paranoia, and other anxiety-like symptoms. It is therefore unsurprising that individuals higher in this trait are more likely to notice, report, and feel distressed by these reactions.

Lastly, agreeableness was found to predict a significant portion of unique variance in the total number of different reactions endorsed, and conscientiousness predicted a significant portion of unique variance in distress associated with adverse reactions to cannabis. Each of these regression coefficients was small and positive, indicating that higher levels of these personality traits were associated with increased levels of these aspects of adverse reactions to cannabis. More specifically, the results indicate that individuals who are more agreeable are more likely to endorse a wider variety of adverse reactions. It is unclear whether this is a response bias (i.e., whether these individuals are simply more likely to agree that they have experienced these reactions). Bivariate correlations between agreeableness and the number of different reactions experienced and between conscientiousness and the distress levels associated with adverse reactions revealed that these personality traits were not significantly correlated with these aspects of adverse reactions to cannabis. This pattern of results suggests that these two personality variables are acting as suppressor variables (i.e., suppressing error variance in other predictors, making them stronger predictors of these outcome variables). As such, these results may be largely spurious and additional research is needed to confirm the validity of these unexpected results.

Limitations to this study primarily relate to the reliance on cross-sectional survey data from a self-selected university student sample primarily comprising white (> 70%) female (> 70%) recreational (> 90%) cannabis users who, on average, used cannabis at a moderate frequency. Therefore, while a university student sample was intentionally sought because this population is known to use cannabis at high rates (SAMHSA 2016), the present results may not generalize to other populations of cannabis users. As such, future research should seek to replicate these findings in more diverse samples. Additionally, while our sample of cannabis users reported using a variety of cannabis use methods, consistent with previous research (Sexton et al. 2016), the majority of the sample (> 80%) reported predominantly using inhalation methods of administration, while less than 15% reported predominantly using an oral route of administration. As such, the present results may not reflect adverse reactions associated with oral or other routes of administration as well as they reflect more traditional inhalation methods. Further, these reactions were retrospectively assessed and therefore may be prone to recall bias. An additional limitation to this study pertains to the fact that the adverse reactions documented in this study were predominantly physiological and psychological. Future research should expand upon these reactions to include a wider range of possible social, emotional, and physical reactions to cannabis intoxication (e.g., dry eyes). Finally, the DASS-21 was used to assess the relationships between negative affect variables (depression, anxiety, and stress) and ARS outcomes. However, the DASS-21 assesses mood over the past week, while the timeframe covered by the ARS is indefinite. As such, it is possible that this study failed to detect relationships between negative affect variables measured by the DASS-21, and ARS outcomes because of the differences in time frame assessed by these two measures.

## Conclusions

The present study provides an expanded list of 26 possible adverse reactions to cannabis, as well as their prevalence, frequency, and average distress ratings, and represents a more comprehensive documentation of possible acute adverse reactions to cannabis, relative to existing literature on the topic. Collectively, results of this study suggest that there are a broad range of possible adverse reactions to cannabis and that many of these reactions may occur with higher prevalence and frequency than past research on more experienced cannabis users has indicated. While some adverse reactions appear to be relatively common (e.g., coughing fits, body humming, and racing heart), the present study revealed that none of these reactions are perceived as severely distressing. This study also illuminated numerous predictors of these adverse reactions, including frequency of cannabis use, symptoms of cannabis use disorder, conformity motives, anxiety sensitivity, and personality. This suggests that some individuals may be slightly more likely than others to experience a variety of adverse reactions to cannabis, or to interpret them as distressing, given differences in their cannabis use patterns and motives, and possibly their personality. However, the set of predictors used in this study only explained between 15 and 25% of the variance in various aspects of adverse reactions to cannabis, suggesting that there are other factors which predict adverse reactions to cannabis that this study did not explore, and that future research should seek to identify. Nevertheless, the results of this study have implications for medical professionals working with cannabis users, as well as individuals working in cannabis retail stores (budtenders) who are frequently asked for advice about cannabis products. Similarly, these findings would be of practical value to less experienced medical and recreational cannabis users, who are likely unaware of the range of possible adverse reactions to cannabis, or who may be at increased risk of experiencing, or feeling distressed by, these reactions.

## Supplementary information

**Additional file 1: Table S1.** Comparisons of Random Responders and Eligible Participants on Each Outcome and Predictor. * indicates *p* < .002 (alpha = 0.5/25 comparisons = .002)

**Additional file 2: Table S2.** Bivariate Correlations Between All Outcome and Predictor Variables and Quantity Variables. * indicates *p* < .001

**Additional file 3.** Adverse Reactions to Cannabis Scale

## Data Availability

The dataset used and analyzed for the current study are available from the corresponding author on reasonable request.

## Footnotes

1 A comparison of random responders and eligible responders is included in Additional file 1: Table S1.

2 This frequency measure was added to the ARS when data collection was partially completed.

3 It is unclear why the Cronbach’s alpha value for Openness to Experience was lower in the present sample than in past research.
