# Supplementary material for: Overbaked: assessing and predicting acute adverse reactions to Cannabis
Source: J Cannabis Res. 2020 Jan 2;2:3. doi: 10.1186/s42238-019-0013-x (PMC7819287; doi:10.1186/s42238-019-0013-x)
Supplement: Supplementary file 1 — Additional file 1: Table S1. Comparisons of Random Responders and Eligible Participants on Each Outcome and Predictor. * indicates p < .002 (alpha = 0.5/25 comparisons = .002) [file 42238_2019_13_MOESM1_ESM.docx]

**Additional file 1 Table S1. Comparisons of Random Responders and Eligible Participants on Each Outcome and Predictor**

| **Predictor** | **Random Responders**  ***M* (SE)** | **Eligible**  **Participants**  ***M* (SE)** | ***t* (SE)** |
| --- | --- | --- | --- |
| # Different Adverse Reactions | 9.10 (0.47) | 7.63 (0.19) | 2.98 (0.49) |
| Overall Frequency of Adverse Reactions | 23.50 (3.43) | 22.01(1.19) | 0.46 (3.24) |
| Overall Mean Distress of Adverse Reactions | 1.38 (0.07) | 1.26 (0.03) | 1.62 (0.07) |
| Daily Sessions of Cannabis Use | -0.02 (0.08) | -0.07 (0.03) | 0.55 (0.08) |
| Frequency of Cannabis Use | 0.02 (0.07) | -0.03 (0.02) | 0.76 (0.07) |
| Age of Onset of Cannabis Use | 0.06 (0.05) | 0.08 (0.02) | -0.34 (0.06) |
| Quantity of Cannabis Use | 0.13 (0.08) | -0.02 (0.03) | 1.92 (0.08) |
| Cannabis Use Disorder | 1.04 (0.07) | 1.02 (0.03) | 0.30 (0.06) |
| Coping Motives | 2.46 (0.09) | 2.22 (0.04) | 2.46 (0.09) |
| Enhancement Motives | 3.10 (0.09) | 3.15 (0.04) | -0.54 (0.10) |
| Social Motives | 2.31 (0.08) | 2.25 (0.03) | 0.69 (0.09) |
| Conformity Motives | 1.58 (0.06) | 1.42 (0.02) | 2.63 (0.06) |
| Expansion Motives | 2.25 (0.09) | 2.11 (0.04) | 1.47 (0.10) |
| Routine Motives | 2.67 (0.08) | 2.53 (0.08) | 1.59 (0.09) |
| Openness to Experience | 2.31 (0.03) | 2.30 (0.01) | 0.29 (0.04) |
| Conscientiousness | 2.35 (0.04) | 2.55 (0.02) | -4.50* (0.05) |
| Extraversion | 2.21 (0.04) | 2.44 (0.02) | -4.87* (0.05) |
| Agreeableness | 2.42 (0.04) | 2.65 (0.02) | -5.31* (0.04) |
| Neuroticism | 2.33 (0.05) | 1.93 (0.02) | 6.87* (0.06) |
| Anxiety Sensitivity | 1.75 (0.06) | 1.48 (0.02) | 4.55* (0.06) |
| Depression | 1.08 (0.06) | 0.68 (0.02) | 6.90* (0.06) |
| Anxiety | 0.92 (0.05) | 0.65 (0.02) | 5.52* (0.05) |
| Stress | 1.20 (0.05) | 0.92 (0.02) | 5.15* (0.05) |
| Age | 20.67 (0.27) | 20.81 (0.12) | 0.03 (0.36) |
| Gender | 1.76 (0.03) | 1.73 (0.01) | 0.82 (0.38) |

* indicates *p* < .002 (alpha = 0.5/25 comparisons = .002)
