# Supplementary material for: Overbaked: assessing and predicting acute adverse reactions to Cannabis
Source: J Cannabis Res. 2020 Jan 2;2:3. doi: 10.1186/s42238-019-0013-x (PMC7819287; doi:10.1186/s42238-019-0013-x)
Supplement: Supplementary file 2 — Additional file 2: Table S2. Bivariate Correlations Between All Outcome and Predictor Variables and Quantity Variables. * indicates p < .001 [file 42238_2019_13_MOESM2_ESM.docx]

**Additional file 2 Table S2: Bivariate Correlations Between All Outcome and Predictor Variables and Quantity Variables.**

| **Predictor** | **1.** | **2.** | **3.** | **4.** | **5.** | **6.** | **7.** | **8.** | **9.** | **10.** | **11.** | **12.** | **13.** | **14.** | **15.** | **16.** |
| --- | --- | --- | --- | --- | --- | --- | --- | --- | --- | --- | --- | --- | --- | --- | --- | --- |
| **1. # Different Reactions** | -- |  |  |  |  |  |  |  |  |  |  |  |  |  |  |  |
| **2. Frequency of Reactions** | .10 | -- |  |  |  |  |  |  |  |  |  |  |  |  |  |  |
| **3. Distress from Reactions** | .45* | .26* | -- |  |  |  |  |  |  |  |  |  |  |  |  |  |
| **4. Daily Sessions** | .03 | -.22* | -.05 | -- |  |  |  |  |  |  |  |  |  |  |  |  |
| **5. Frequency** | .01 | -.39 | -.19 | .66* | -- |  |  |  |  |  |  |  |  |  |  |  |
| **6. Age of Onset** | -.11* | .07 | -.09 | -.23* | -.19* | -- |  |  |  |  |  |  |  |  |  |  |
| **7. Quantity of Cannabis** | -.02 | -.11 | -.03 | .55* | .48* | -.25* | -- |  |  |  |  |  |  |  |  |  |
| **8. Quantity of Concentrate** | .01 | .05 | -.06 | .32* | .28* | -.11 | .23* | -- |  |  |  |  |  |  |  |  |
| **9. Quantity of Edible** | .05 | .05 | -.08 | .24* | .25* | -.12 | .35* | .20* | -- |  |  |  |  |  |  |  |
| **10. Cannabis Use Disorder** | .15* | -.22* | .01 | .53* | .69* | -.14* | .42* | .16* | .22* | -- |  |  |  |  |  |  |
| **11. Coping Motives** | .10* | -.14 | -.11* | .28* | .43* | -.08 | .22* | .21* | .05 | .42* | -- |  |  |  |  |  |
| **12. Enhancement Motives** | .08 | -.23* | -.22* | .30* | .55* | -.13* | .19* | .25* | .21* | .37* | .47* | -- |  |  |  |  |
| **13. Social Motives** | .07 | -.11 | -.13 | .19* | .28* | -.07 | .18* | .16* | .13 | .27* | .49* | .58* | -- |  |  |  |
| **14. Conformity Motives** | .18* | .13 | .22* | -.11 | -.17* | -.01 | -.06 | -.02 | -.03 | .05 | .12* | -.05 | .29* | -- |  |  |
| **15. Expansion Motives** | .12* | -.16 | -.13* | .26* | .41* | -.12* | .25* | .14 | .21* | .39* | .51* | .49* | .51* | .20* | -- |  |
| **16. Routine Motives** | .13* | -.22* | -.13* | .39* | .58* | -.20* | .29* | .17* | .12 | .51* | .67* | .61* | .45* | .08 | .56* | -- |
| **17. Openness to Experience** | .09 | -.08 | -.08 | .12* | .17* | .02 | .05 | .04 | .03 | .09 | .14* | .17* | .09 | -.12* | .24* | .10* |
| **18. Conscientiousness** | -.08 | -.04 | .05 | .01 | -.10 | .03 | -.03 | .04 | -.003 | -.11* | -.16* | -.04 | -.12* | -.17* | -.07 | -.12* |
| **19. Extraversion** | -.02 | -.04 | -.05 | -.04 | -.01 | .02 | -.06 | -.001 | .07 | -.07 | -.21* | .11* | -.003 | -.07 | .03 | -.05 |
| **20. Agreeableness** | -.04 | -.03 | -.004 | -.07 | -.04 | .13* | -.12* | -.09 | -.05 | -.12* | -.17* | .03 | -.10* | -.18* | -.09 | -.14* |
| **21. Neuroticism** | .20* | .10 | .10 | .05 | .09 | .01 | .02 | .06 | -.03 | -.14* | .41* | .07 | .18* | .16* | .13* | .22* |
| **22. Anxiety Sensitivity** | .24* | .11 | .22* | -.03 | -.02 | -.01 | -.05 | .04 | .05 | .02 | .28* | .05 | .17* | .26* | .14* | .17* |
| **23. Depression** | .17* | .06 | .07 | .09 | .05 | -.03 | .05 | .03 | .02 | .13* | .38* | .06 | .16* | .22* | .15* | .19* |
| **24. Anxiety** | .22* | .04 | .08 | .12* | .11* | -.03 | .05 | .07 | -.01 | .13* | .36* | .08 | .20* | .24* | .21* | .24* |
| **25. Stress** | .20* | .12 | .08 | .02 | -.01 | -.01 | -.02 | .02 | -.07 | .03 | .32* | .04 | .14* | .18* | .13* | .19* |
| **26. Age** | .03 | .15 | .05 | .05 | -.07 | .11* | .004 | -.07 | -.05 | -.01 | -.05 | -.17* | -.18* | .08 | -.07 | -.06 |
| **27. Gender** | .10* | .10 | .11* | -.09 | -.07 | .08 | -.17* | .06 | -.16 | -.13* | -.09 | -.02 | -.03 | -.10 | -.08 | -.02 |

| **Predictor** | **17.** | **18.** | **19.** | **20.** | **21.** | **22.** | **23.** | **24.** | **25.** | **26.** | **27.** |
| --- | --- | --- | --- | --- | --- | --- | --- | --- | --- | --- | --- |
| **17. Openness to Experience** | -- |  |  |  |  |  |  |  |  |  |  |
| **18. Conscientiousness** | .06 | -- |  |  |  |  |  |  |  |  |  |
| **19. Extraversion** | .07 | .33* | -- |  |  |  |  |  |  |  |  |
| **20. Agreeableness** | .11* | .32* | -.39* | -- |  |  |  |  |  |  |  |
| **21. Neuroticism** | .05 | .39* | -.42* | -.31* | -- |  |  |  |  |  |  |
| **22. Anxiety Sensitivity** | .02 | -.19* | -.16* | -.21* | .53* | -- |  |  |  |  |  |
| **23. Depression** | .06 | -.36* | -.40* | -.31* | .68* | .46* | -- |  |  |  |  |
| **24. Anxiety** | .05 | -.25* | -.25* | -.24* | .58* | .56* | .66* | -- |  |  |  |
| **25. Stress** | .06 | -.23* | -.25* | -.27* | .67* | .50* | .72* | .73* | -- |  |  |
| **26. Age** | .07 | .10* | -.14* | -.03 | -.01 | -.08 | .03 | -.06 | .04 | -- |  |
| **27. Gender** | .04 | .02 | -.04 | -.16* | .21* | .16* | .11* | .12* | .20* | .001 | -- |

* indicates *p* < .001
