# Supplementary material for: Overbaked: assessing and predicting acute adverse reactions to Cannabis
Source: J Cannabis Res. 2020 Jan 2;2:3. doi: 10.1186/s42238-019-0013-x (PMC7819287; doi:10.1186/s42238-019-0013-x)
Supplement: Supplementary file 3 — Additional file 3. Adverse Reactions to Cannabis Scale [file 42238_2019_13_MOESM3_ESM.docx]

**Additional file 3:**

**Adverse Reactions to Cannabis Scale**

**Instructions:** We are interested in whether you have experienced any of the following adverse reactions to acute cannabis intoxication. When providing your answers please only consider times when you were high on cannabis and when the symptom was a direct result of cannabis.

While under the influence of cannabis, have you experienced any of the following?

Anxiety Yes/No

Panic Attack Yes/No

Feeling out of control Yes/No

Migraine/Headache Yes/No

Vomiting Yes/No

Nausea Yes/No

Cold Sweats Yes/No

Hot Flash Yes/No

Tunnel Vision Yes/No

Dizzy Yes/No

Light headed/head rush Yes/No

Off balance/unsteady Yes/No

Seeing black spots Yes/No

Fainting/passing out Yes/No

Racing heart Yes/No

Heart palpitations/arrhythmia Yes/No

Chest/lung discomfort Yes/No

Trouble breathing Yes/No

Coughing fit Yes/No

Paranoia Yes/No

Auditory hallucinations Yes/No

Visual hallucinations Yes/No

Other hallucinations Yes/No

Dissociation (i.e. feeling disconnected from self or reality) Yes/No

Numbness Yes/No

Feelings of body humming or vibrating Yes/No

*Display these questions for any symptoms for which [Yes] is selected above:*

Approximately what percentage of the time that you use cannabis do you experience ____[symptom name (e.g. Anxiety]?

On average, how distressing was experiencing ______ [symptom name (e.g. Anxiety] while under the influence of cannabis?

0 = Not at all distressing; 1 = Mildly distressing; 2 = Moderately distressing; 3 = Quite distressing 4 = Severely distressing
